# Genome-wide diversity analysis to infer population structure and linkage disequilibrium among Colombian coconut germplasm

**DOI:** 10.1038/s41598-022-07013-w

**Published:** 2022-02-22

**Authors:** Jorge Mario Muñoz-Pérez, Gloria Patricia Cañas, Lorena López, Tatiana Arias

**Affiliations:** 1 Laboratorio de Biología Comparativa, Corporación Para Investigaciones Biológicas, Cra. 72 A No. 78B 141, Medellín, Antioquia Colombia; 2 Unidad de Sanidad y Control Biológico, Corporación Para Investigaciones Biológicas, Cra. 72 A No. 78B 141, Medellín, Antioquia Colombia; 3 Present Address: Marie Selby Botanical Gardens, Downtown Sarasota Campus, 1534 Mound Street, Sarasota, FL 34236 USA

**Keywords:** Evolution, Plant sciences

## Abstract

Genetic diversity and relatedness of accessions for coconut growing in Colombia was unknown until this study. Here we develop single nucleotide polymorphisms (SNPs) along the coconut genome based on Genotyping by Sequencing (GBS) with the goal of analyze the genetic diversity, population structure, and linkage disequilibrium (LD) of a diverse coconut panel consisting of 112 coconut accessions from the Atlantic and Pacific coasts of Colombia. A comprehensive catalog of approximately 40,000 SNPs with a minor allele frequency (MAF) of > 0.05 is presented. A total of 40,614 SNPs were found but only 19,414 anchored to chromosomes. Of these, 10,338 and 4606 were exclusive to the Atlantic and Pacific gene pools, respectively, and 3432 SNPs could differentiate both gene pools. A filtered subset of unlinked and anchored SNPs (1271) showed a population structure at K = 4, separating accessions from the Pacific and Atlantic coasts that can also be distinguished by palm height, as found in previous studies. The Pacific groups had a slow LD decay, low Fixation Index (Fst) and low nucleotide diversity (π), while the Atlantic group had slightly higher genetic diversity and faster LD decay. Genome-wide diversity analyses are of importance to promote germplasm conservation and breeding programs aimed at developing new cultivars better adapted to the region.

## Introduction

Coconut (*Cocos nucifera*) is cultivated extensively in all tropical countries around the world and considered one of the most important plant species to guarantee the survival of humankind^1^. Origins of cultivated coconut are difficult to trace strictly by morphology because coconuts lack clear domestication traits due to the widespread nature of admixed populations^2^. Two distinctively different forms of the coconut fruit, known as "Niu kafa” and “Niu vai”—Samoan names for traditional Polynesian varieties—are known. The Niu kafa form is triangular and oblong with a large fibrous husk. The Niu vai form is rounded and contains abundant sweet coconut “water” when unripe. Coconut palms are also classified by their height, “tall” type palms have evolved naturally and were disseminated by either the Pacific or the Atlantic Ocean currents. Tall palms come from the Indian and Pacific Oceans and are generally cross-pollinated, having long stems and late bearing fruit, and can be of the “Niu kafa” or “Niu vai” type^1,3^. The “dwarf” type palms are recognized for being self-pollinated, and displaying important domestication features such as short stems, high productivity in terms of fruit production, and low genetic variability^4^. Both palm types gave rise to a vast number of coconut populations of pantropical distribution that are poorly characterized at the genomic level, and that are generally identified based on variable morphological and agronomic traits, which are not apparent in juvenile phases^5–7^.

Coconut palms are monoic, having both male and female flowers on the same inflorescence, perennial diploids (2n = 32) and the sole species of the genus *Cocos*. Draft genomes have been published suggesting a genome size of about 1.93 Gb (dwarf coconut) to 2.4 Gb (tall Hainan coconut) and a complex genome structure composed of 50–70% of repetitive sequences, chromosome fractionation and duplication followed by rearrangements^2,4,8,9^. Many studies have been conducted to characterize the genetic diversity of coconut collections, and to understand coconut cultivation history^1,10,11^. However, northern South America is an underrepresented region in these studies. RFLP^12^, microsatellite^1,10,12,13^, and AFLP^14,15^ were used to understand coconut genetic diversity. High levels of genetic differentiation between populations of Pacific and Indo-Atlantic coastal regions have been proposed by most authors, suggesting coconut have a long-standing evolutionary history and were brought into cultivation independently in each of these regions. Evidence coming from sources such as microsatellites^1^ and written historical records^16^ suggest *C. nucifera* was introduced to Colombia. Coconut Pre-Columbian introduction from Southeast Asia to the central and south American Pacific coast covered a region spanning from Costa Rica to Peru^16^. Atlantic coconut populations probably came from India^1^ and were introduced directly or indirectly to Colombia after Vasco da Gama^17^. Dwarf coconut has been introduced to Colombia more recently and on both coasts^1^.

High-throughput sequencing has enabled the discovery of single nucleotide polymorphisms (SNPs) throughout the genome, greatly increasing power for detecting neutral and adaptive patterns of variation^18,19^. Genotyping by sequencing (GBS) is considered an efficient and economical method to quickly discover SNPs among several individuals simultaneously^18,20^, allowing plant breeders to use these resources for crop improvement. Such an approach has benefited many crops like watermelon^21^, cowpea^22^, rice^23^, and spinach^24^, among many others. The development of SNPs markers in coconut represents potential for application of molecular breeding techniques through marker-assisted selection (MAS) and association mapping.

Despite the central importance of coconut as a widely distributed and cultivated palm around the world, little is known about the genomic diversity of this species in Colombia because coconut is not a central part of agriculture in the country. Here we tested if there is genetic differentiation within the country between samples from the Pacific and Atlantic coast. This study aims to: (1) identify SNPs at the genome level, (2) investigate the genetic diversity and population structure, and (3) characterize the linkage disequilibrium (LD) in coconut growing in Colombia. We present a comprehensive catalogue of approximately 40,614 SNPs in coconut, based on the GBS method of 112 accessions of unknown genetic origin. Our work provides a better understanding of the genetic diversity and population structure of coconut populations in Colombia.

## Materials and methods

### Plant materials

A total of 112 coconut mature individuals were selected from the main coconut producing departments on the Atlantic and Pacific coasts of Colombia. Most sites in both coasts covered some production sites, only a natural site in the pacific coast was screened. Two sampling sites in the Atlantic coast included: (1) 26 accessions from Córdoba, municipalities of Puerto Escondido (locations: Mucuna, El Paraiso, La Union and Puerto Alegre) and Moñitos (locations: Pueblito, La Rada, Behiacoita and El Destino). (2) 27 accessions from Antioquia, municipalities of San Juan de Urabá (locations: Uveros y La Balsilla) and Arboletes (locations: La Fortaleza y El Destino). Three sites were in the Pacific coast: (1) 25 accessions from Nariño, municipality of Tumaco (locations: San Jose del Guayabo, Tablón Dulce, Chagui, Buenos Aires, Rosario and Gualayo). (2) 27 accessions from Cauca, municipality of Guapi (locations: Playa Blanca, Quiroga, Preba, Obregón) and (3) only seven natural accessions from Chocó, municipality of Nuquí (location: Coquí). Coconut palms are a combination of adventive admixed populations and perennial crops with a worldwide distribution. Selection of accessions was made trying to cover all different cultivated coconut varieties and adventive coconut palms present in each zone. There was not available information about specific cultivars grown in each zone, so individuals were geo-referenced and marked in the field (Table S1).

### DNA extraction, library preparation and sequencing

A sample of fresh leaf tissue was collected and preserved in silica gel for each palm. DNA extraction was performed from leaf tissue collected in the field. A combination of CTAB (Hexadecyl Trimethyl Ammonium Bromide) extraction method^25^ with Epoch Life Science (Epoch Life Science Inc. Missouri, Texas, USA) purification columns were used to extract and purify DNA. DNA quality was determined by spectrophotometry using Nanodrop 2000 (Thermo Fisher Scientific, Waltham, Massachusetts, USA) and DNA concentration through fluorometry using Qubit 3.0 (Life Technologies Carlsbad, California, USA).

Library construction and sequencing was outsourced with LGC Genomics (Berlin, Germany). Initially a pilot experiment using 12 samples (three accessions and four populations: two from the Pacific coast and two from the Atlantic coast) was performed; to find out which set of restriction enzymes work better to recover more Single Nucleotide Polymorphisms (SNPs) in coconut. Between 100 and 200 ng of genomic DNA was digested using 2 units of the *MslI* restriction enzyme (New England Biolabs, Ipswich, Massachusetts, USA) and using one unit of NEB4 buffer in 20 μl of volume, incubated for two hours at 37 °C. The restriction enzyme was inactivated by incubation at 80 °C for 20 min. The same procedure was used for double digestion with the *PstI-MspI* enzymes (New England Biolabs, Ipswich, Massachusetts, USA). Enzyme *MslI* was chosen for this work because many more SNPs were recovered (Table S2).

For the ligation reaction, 10 μl of each restriction digest were transferred to a new 96 well PCR plate, mixed on ice first with 1.5 μl of one of 96 inline-barcoded forward blunt adaptors (pre-hybridized, concentration 5 pmol/μl), followed by addition of 20 μl ligation master mix containing 15 μl NEB Quick ligation buffer (New England, Biolabs, Ipswich, Massachusetts, USA), 0.4 μl NEB Quick Ligase (New England, Biolabs, Ipswich, Massachusetts, USA), and 7.5 pM pre- hybridized common reverse blunt adaptor. Ligation reactions were incubated for 1 h at room temperature, followed by heat inactivation for 10 min at 65 °C. For library purification, all reactions were diluted with 30 μl TE 10/50 (10 mM Tris/HCl, 50 mM EDTA, pH: 8.0) and mixed with 50 μl Agencourt® XP beads (Beckman and Coulter, Indianapolis, USA), incubated for 10 min at room temperature and placed for five min on a magnet to collect the beads. The supernatant was discarded, and the beads were washed two times with 200 μl 80% ethanol. Beads were air dried for 10 min and libraries were eluted in 20 μl Tris Buffer (5 mM Tris/HCl pH: 9.0).

For library amplification, 10 μl of each of the 96 Libraries were separately amplified in 20 μl PCR reactions using MyTaq™ (Meridian Bioline, Memphis, Tennessee, USA) and standard Illumina TrueSeq™ amplification primers (Illumina, San Diego, California, USA). Cycle number was limited to 14 Cycles. Pooling and cleanup of GBS libraries was done using 5 μl from each of the 96 amplified libraries. PCR primer and small amplicons were removed by Agencourt® XP bead purification (Beckman Coulter Life Sciences, Indianapolis, USA) using one volume of beads. The PCR enzyme was removed by an additional purification on MinElute® columns (Qiagen, Marylan, USA). The pooled Library was eluted in a final volume of 20 μl Tris Buffer (5 mM Tris/HCl pH: 9).

Normalization was done using Trimmer Kit (Evrogen, Moscow, Russia). One μg pooled GBS library in 12 μl was mixed with a four μl 4 × hybridization buffer, denatured for three min at 98 °C and incubated for five hours at 68 °C to allow reassociation of DNA fragments. Twenty μl of 2 × DSN master buffer was added, and the samples were incubated for 10 min at 68 °C. One unit of DSN enzyme (1 U/μl) was added and the reaction was incubated for another 30 min. Reaction was terminated by the addition of 20 μl DSN Stop Solution, purified on a Qiagen ® Column (Qiagen, Marylan, USA) and eluted in 10 μl Tris Buffer (5 mM Tris/HCl pH: 9). The normalized library pools were re-amplified in 100 μl PCR reactions using MyTaq™ (Meridian Bioline, Memphis, Tennessee, USA). For each pool, a different i5-Adaptor primer was used to include i5-Indices into the libraries, allowing parallel sequencing of multiple libraries on the Illumina NextSeq 500 sequencer (Illumina, San Diego, California, USA). Cycle number was limited to 14 cycles. The GBS libraries were size selected on Blue Pippin (Sage Science, Massachusetts, USA), followed by a second size selection on an UltraPure™ Low Melting Point Agarose (LMP) agarose gel (Thermo Fisher Scientific, Massachusetts, USA), removing fragments smaller than 300 bp and those larger than 400 bp. Sequencing was performed on an Illumina NextSeq® 500 (Illumina, San Diego, California, USA) using V2 Chemistry (300 cycles).

### Data processing

For data pre-processing, filtering of restriction enzyme site at 5' end of reads was performed and reads with 5' end not matching the restriction enzyme site were discarded. Quality trimming was performed by removing adapter sequences, reads containing Ns, and trimming reads at 3'-end to get a minimum average Phred quality score of 20 over a window of ten bases. Reads with final length < 20 bases were discarded. A subsampling (evenly across the complete FASTQ files) of quality-trimmed reads was performed to 1.5 million read pairs per sample. FastQC reports (Andrews 2008) were prepared for all FASTQ files and read counts were recorded. All pipelines used here can be found at https://github.com/TheAriasLab/POPULATIONS_GENOMICS.

For the Stacks pipeline version 2.5^26^, Bowtie2 version 2.4.2^27^ was used to index the “Hainan Tall Coconut” draft reference genome^28^ and to align reads to the reference genome index. Samtools version 1.11^29^ was used to compress SAM files and to convert them to their binary version (BAM). The Stacks script “*populations*” was implemented to filter and identify Single Nucleotide Polymorphisms (SNPs) according to a minimum percentage of individuals (80%) in a population required to process a locus, a minimum allele frequency (0.05) of a SNP to be considered, and a maximum observed heterozygosity (0.70) of a SNP to be considered. Total recovered SNPs included those anchored to chromosomes and those in scaffolds. The software Structure^30^ requires unlinked markers, to avoid misleading results, linked SNPs were filtered (LD > 0.2). Only SNPs anchored to chromosomes were included in this analysis.

### Population statistics

The Fst index (Holsinger et al. 2009) was calculated between the Atlantic and Pacific coast gene pools using the method of Weir and Cockerham^31^ with pairwise Fst on a per site basis. For SNPs anchored to chromosomes, Fst values for each site correspond to the mean Fst value between the site and all the other sites across the genome. Nucleotide diversity (π)^32^ was calculated on a per site basis and averaged for each data set (Atlantic and Pacific) and Tajima’s D^33^ was calculated on a 100 k sliding window on each data set (Atlantic and Pacific). All calculations were carried out using vcftools version 0.1.15^34^.

### Genetic structure of populations

A Bayesian method in Structure version 2.3.4^30^ was used to identify genetic structure of populations with an admixture model, a burn-in of 25,000, and several MCMC replicates after burn-in of 250,000 (112 individuals and SNPs anchored to chromosomes and filtered by LD > 0.2). Runs were performed from k = 1 to k = 6, assuming coconut populations from Colombia could show population substructure based on a global divide between Atlantic and Pacific populations^1^ and further substructures within each coast. Each run was repeated 10 times for a total of 60 runs per test. The method of Evanno et al.^35^ and associated R scripts were used to calculate delta K as a measure that best describes the number of clusters in the data. Permutation of runs was done using Clumpp 1.1.2^36^ and visualization of the data was done using R scripts. Recent hybrids were detected using Snapclust from the Adegenet R package V2.1.3.^37^ and the kinship coefficient Identity By Descent (IBD) was calculated using PLINK v1.90b5.2, Method of Moment (MoM)^29^.

### Linkage disequilibrium analysis

To calculate LD PopLDdecay version 3.41^38^ was used for each group found in the genetic Structure analysis. Set parameters were -Het 0.9 -Miss 0.1 -MAF 0.1. SNPs around 120 Mb were exclusively used for LD calculations. Mean LD for genomic bins was calculated using PopLDdecay with the following parameters -bin1 2000 -bin2 1,000,000 -break 600 for Pacific group 1, -bin1 2000 -bin2 3,000,000 -break 2000 for Pacific group 2 and -bin1 2000 -bin2 1,000,000 -break 600 for group “both coasts” and Atlantic. To calculate LD decay and half decay we fit the data to the Hill and Weir^39^ model using R.

### Phylogenetic networks

To understand relationships and distances between samples, we used SplitsTree version 5^40^ to infer a phylogenetic network with the Neighbor-net algorithm.

### Preprint

A previous version of this paper has been published as a preprint here: (Vitti et al., 2013). This past version differs from the current version because a new and updated coconut genome assembly to chromosomes was published before the past version was published. While the main core of results is very similar, we have done a series of analysis and figures that differ significantly from the previous version.

### Ethical approval

We have all collection permits required by the Colombian government to collect leaves samples of mature individuals for DNA extractions: Autoridad Nacional de Licencias Ambientales (ANLA)- 8 de Octubre 2015, Resolución Número 1263. All methods were carried out in accordance with relevant guidelines and regulations.

## Results

### Genome-wide SNPs discovery

GBS analysis was performed in samples from 112 specimens of adventive and commercially cultivated coconut from the main coconut producing areas in both the Atlantic (Antioquia and Córdoba) and Pacific (Nariño, Cauca and Chocó) Colombian coasts. In total, approximately 182 million of raw reads were obtained in this study (NCBI Bioproject PRJNA579494, NCBI SRA Accessions SRR10345275-SRR10345401), from which 168 million could be aligned to the assembled coconut reference genome (VOII00000000.1^28^), resulting in an average mapping rate of 92.47%. Initially, 40,614 SNPs were obtained with a frequency of depth above three (Fig. S1), however after filtering those loci that anchored to chromosomes, the dataset resulted in a total of 19,414 SNPs. For population Structure inference and LD calculations anchored SNPs were filtered for LD > 0.2 resulting in 1271 SNPs.

**Figure 1:**
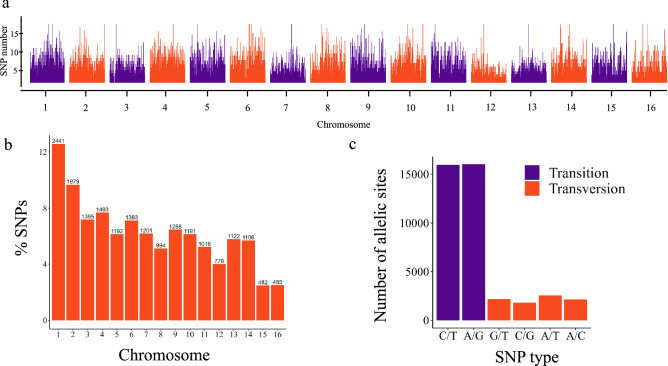
Identification of 19,414 single nucleotide polymorphisms (SNPs) obtained from the genotyping of 112 coconut accessions. (**a**) Distribution of SNP density along Hainan Tall coconut genome. (**b**) Proportion of SNPs in each chromosome. (**c**) Transversion/transition ratio. Figure produced in R v.4.0 (2020)^56^.

SNPs were evenly distributed throughout the genome (Fig. 1a). The mean number of SNPs per chromosome was 1213, ranging from 482 to 2441 SNPs on the fifteen and first chromosomes, respectively. The number of SNPs had a strong positive correlation with the physical chromosome length (r = 0.95, p < 0.01). 12.57% of SNPs were in chromosome one, and 9.67% in chromosome two, while chromosomes 15 and 16 had the lowest percentage of SNPs in the genome with 2.48% and 1.47% respectively (Fig. 1b). Transitions (78.72%) were more frequent than transversions (21.27%) in terms of polymorphisms, resulting in a transition/transversion ratio of 3.70 (Fig. 1c). Percentages of A/G and C/T transitions were similar (39.41% and 39.32%, respectively), as were those of polymorphism due to A/T, G/T, A/C and G/C transversions (6.27%, 5.36%, 5.24%, and 4.41%, respectively).

### Estimation of relatedness and population structure

**Figure 2:**
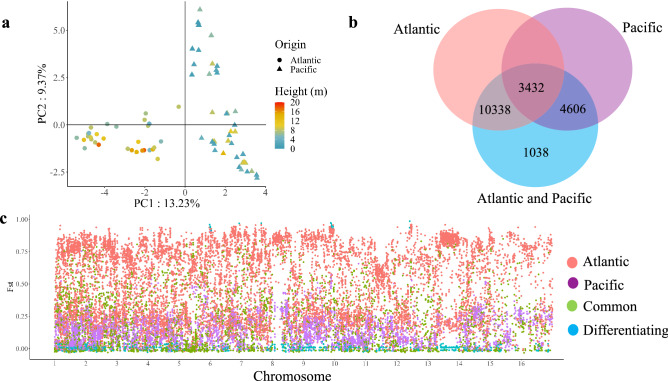
Genetic differentiation between Colombian Atlantic and Pacific coast coconut gene pools. (**a**) Principal component analysis of 99 accessions from the Atlantic and Pacific coast including different commercial groups. (**b**) Venn diagram of the total set of SNPs (19,414) and SNPs belonging to the Atlantic and Pacific groups. (**c**) Distribution of the Fst values of each SNP.

Population genetic structure was analyzed after pruning for linkage disequilibrium (LD). 1271 unlinked SNPs with *r*^2^ < 0.2 of a total of 19,414 were kept for genetic diversity and structure analysis. An initial principal component analysis (PCA) showed that accessions from the Atlantic coast formed a group, whereas accessions from the Pacific coast were divided into two distinct groups (Fig. 2a).

**Figure 3:**
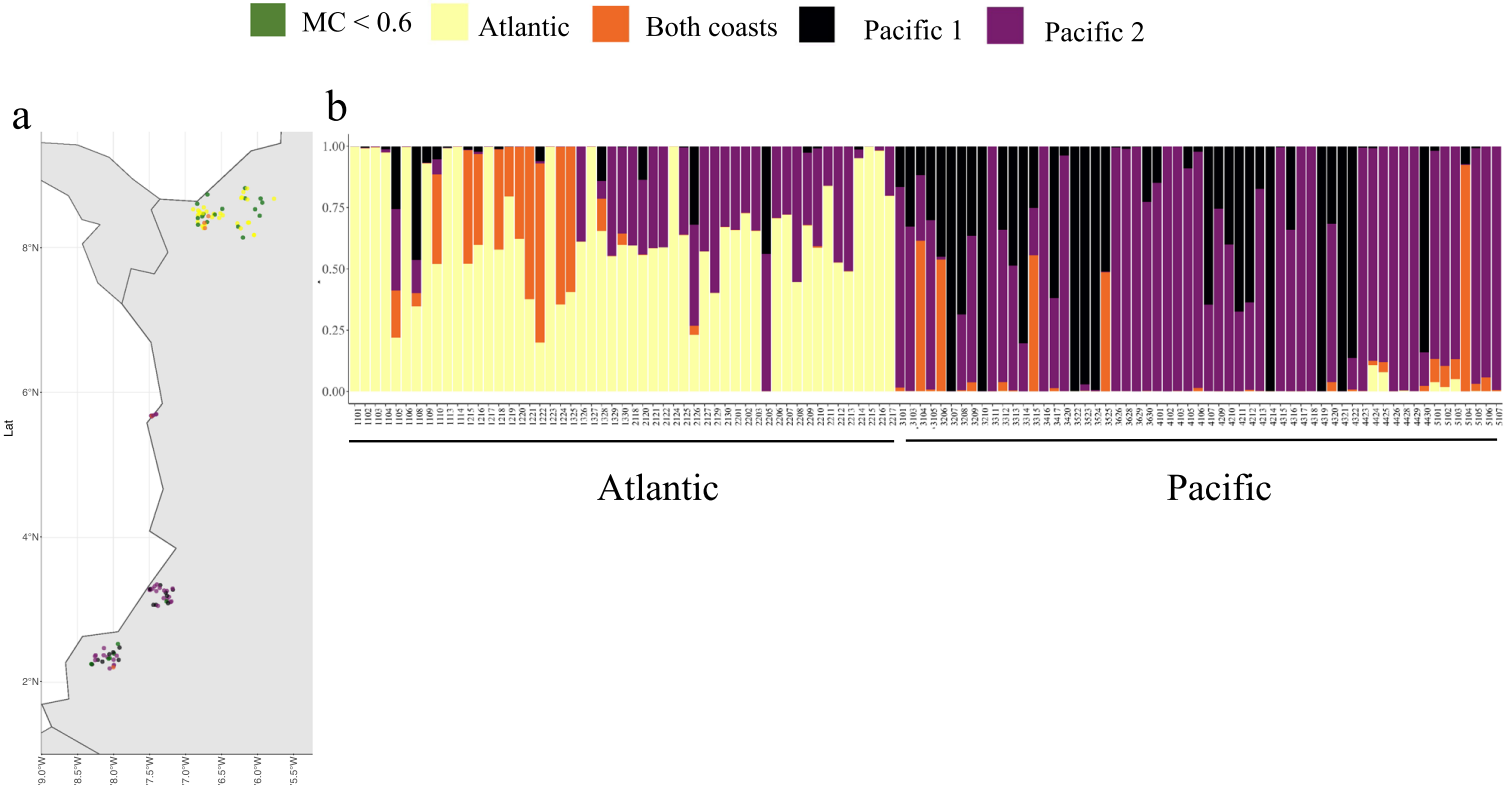
Analysis of the population structure using 112 accessions belonging to the Colombian coconut diversity panel. (**a**) distribution of genotypes along each Colombian coast. (**b**) Structure analysis with K = 4**.**

Gene pools were also determined using a Bayesian population structure analysis based on the ΔK^35^ criterion. Structure analyses were performed using K values ranging from k = 2 to k = 6. The number of groups with the highest value of ΔK was four (Fig. 3, Figs. S2, S3). A membership coefficient (≥ 0.6) was estimated for each accession to determine whether each accession was admixed or could be assigned to a specific genetic structure group. 75.89% of the accessions could be assigned to a specific group, and 27 accessions were categorized as admixed. Admixed accessions comprised what had resulted from hybridization between two or more of the four groups found with the Structure software. Eleven recent hybrids were detected using Snapclust, all of them in present the Atlantic coast, the parental populations were both from the Pacific and Atlantic gene pools (Fig. S4).

To further understand the genetic relationships among accessions, a pedigree analysis was performed on germplasm collected here. Identity-By- Descent (IBD) analysis was done across all accessions. A pairwise IBD near zero was observed among most accessions from the Pacific coast of Colombia with several exceptions (Fig. S5). While for most of the accessions from the Atlantic IDB values range between 0.2 and 0.3, suggesting little to none first-degree relatedness (siblings, parent-offspring, etc.) among most accessions analyzed here (Fig. S5). Atlantic coast accessions formed a clearly distinguished cluster, and Pacific coast accessions formed two distinct clusters (Fig. 3a). Based on the membership coefficient (≥ 0.6) a fourth group was present in both coasts (Fig. 3). Using all SNPs anchored to both chromosomes and scaffolds (19,414), and after removal of accessions originating on the Atlantic coast, 8,038 SNPs with MAF > 0.05 could be identified in the remaining 51 accessions with an origin in the Pacific coast (Fig. 2b).

### Genetic differentiation between Pacific and Atlantic gene pools

Using SNPs anchored chromosomes (19,414). Both gene pools shared 3,432 SNPs, whereas 4606 and 10,338 SNPs were unique to Pacific and Atlantic accessions (Fig. 2b), respectively. Mean pairwise fixation index (Fst) for each of these SNPs groups was 0.2, 0.16, and 0.47 respectively (Fig. 2c). A total of 1038 highly differentiating SNPs were detected in Atlantic and Pacific coast accessions, with a mean Fst of 0.54 (Fig. 2c). Mean Fst for Atlantic and Pacific coast accessions was 0.35 when all SNPs were included (19,414). Atlantic coast accessions showed slightly greater mean nucleotide diversity (π = 0.34) than Pacific coast accessions (π = 0.32). Both gene pools showed positive Tajima’s D values, having Pacific coast accessions a slightly greater value (D = 1.12) than Atlantic coast accessions (D = 1.09). The average weighted Fst was 0.50.

### Genetic differentiation among genotypes identified using Structure

**Figure 4:**
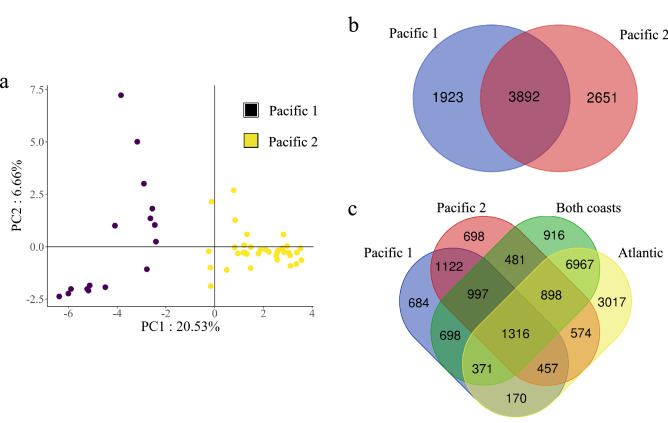
Principal component analyses and Venn diagrams. (**a**) Principal component analysis of 51 accessions of coconuts originated in the Pacific coast. (**b**) Venn diagram for the different sets of SNPs related to the genetic structure found in the Pacific coast group. (**c**) Venn diagram for the different sets of SNPs related to the structure found in all accessions.

As seen in the PCA, in the Bayesian analysis of population structure (Evanno test showed the highest delta k, k = 4, Fig. S2), accessions originating on the Pacific coast were also segregated into two main genetic groups (Figs. 2a, 3a, b, 4a). Using SNPs anchored chromosomes (19,414), the separation of Pacific coast accessions into groups Pacific 1 and Pacific 2 had 1,923 and 2,651 SPNs exclusive to each group, and 3,892 SNPs in common between both (Fig. 4b). When comparing all groups, Atlantic coast accessions showed the highest number of SNPs (6,967) (Table 1, Fig. 4c.). The Pacific 2 group had the lowest π (0.24), whereas π values of the Atlantic and Pacific 1 groups were similar (0.34 and 0.33, respectively) (Table 1, Fig. 4c). According to the Fst, the Atlantic and Pacific 1 groups were the most different, with an Fst value of 0.57, whereas comparisons between the Atlantic and the group that was present in both coasts yielded the lowest Fst value (0.32). Tajima’s D values were all positive in relation to the genotypes observed in the population structure analysis (Table 1).

**Table 1:** Nucleotide diversity (π), Tajima’s D and weighted Fst estimated in the Colombian coconut diversity panel in relation to different genotypes identified.

	N	SNPs	π	D	Fst		
**Origin**					**Pacific**		
Atlantic	29	1377	0.337	0.947	0.501		
Pacific	51	8038	0.324	1.124			

					Pacific 2	Atlantic	Both coasts
**Genotypes**							
Pacific 1	16	5815	0.328	0.510	0.537	0.568	0.488
Pacific 2	35	6543	0.239	0.211		0.529	0.478
Atlantic	29	1377	0.337	0.947			0.319
Both Coasts	27	16,629	0.373	0.711			

N number of accessions, SNPs number of SNPs, π nucleotide diversity, D Tajima’s D statistics (Weir and Cockerham 1984).

### Linkage disequilibrium

**Figure 5:**
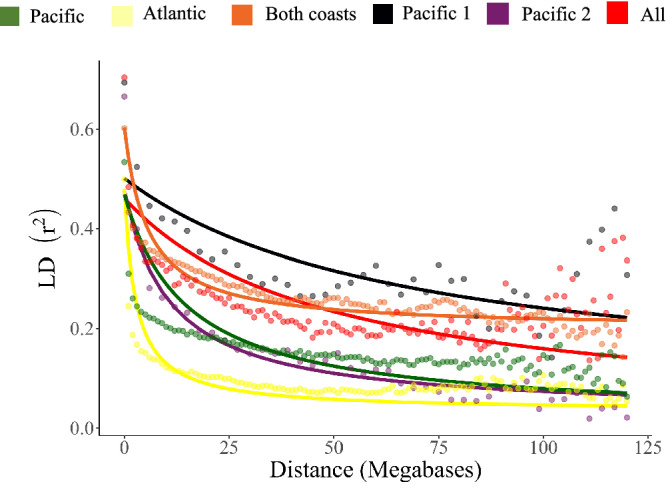
Linkage disequilibrium (LD) graph of coconut grown in northern South America. LD is determined by squared correlations of allele frequencies (r^2^ < 0.3) against distance between polymorphic sites.

LD decay and half-decay distances were calculated for the whole genome including SNPs anchored to chromosomes (19,414), and population structure genotypes. LD decay values observed for Pacific coast accessions was slower (17 Mb) than for Atlantic coast accessions (24 Mb). LD < 0.1 for distances > 16 Mb for Atlantic coast accessions and > 71 Mb for Pacific coast accessions was observed (Fig. 5).

### Phylogenetic networks

**Figure 6:**
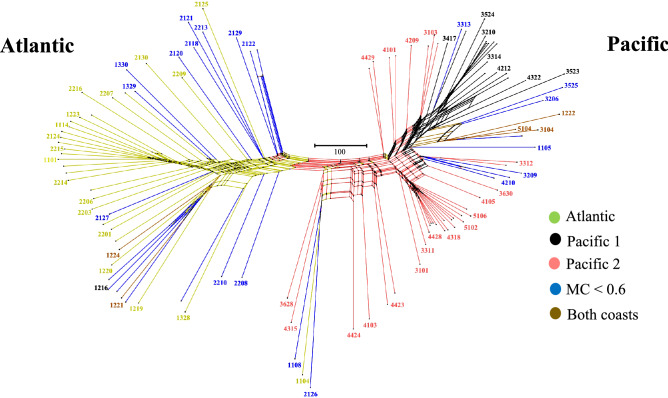
NeighborNet network shows reticulate genetic relationship between accessions. Color coding corresponds to the STRUCTURE clusters of Fig. 3. A scale is shown inset.

A phylogenetic network also revealed accessions from the Atlantic coast to be separated from Pacific coast accessions. Within the Pacific coast group, a smaller well-defined cluster was identified (Fig. 6).

## Discussion

Reduced representation libraries from high-throughput sequencing allowed analysis of SNPs throughout the *C. nucifera* genome, providing both neutral and putatively selected markers. Genotypes were successfully obtained for 112 samples from five different geographical regions in Colombia. A large genome variation dataset for coconut grown in Colombian coastal regions is provided here. The genetic population structure inferred in this study not only supports the hypothesis that there is a strong genetic break between Atlantic and Pacific coast accessions in Colombia but also provides finer structures within the Pacific coast group. This corroborates previous evidence for worldwide genetic diversity of coconut^1^. Results produced here will assist future genome-wide association studies for determining genomic regions or genes associated with several economically important traits.

Here the use of *MslI* restriction enzyme was found to be more effective in GBS of coconut than the double restriction enzymes (*PstI-MspI)* used in a pilot experiment. Coconut genome exhibited a higher number of *MslI* restriction sites, allowing the identification of thousands of markers spaced unevenly throughout the genome (Table S2). Palm studies using a GBS approach have used one of these^41^ or both restriction enzymes^42^. Sequence variations between ‘Hainan Tall’ and ‘Catigan Green Dwarf’ (CATD) genomes included 57,872 SNPs in intergenic regions, 21,066 in genic regions and 5552 in exonic regions^4,9^. This study using GBS recovered 70.18% of the total number of SNPs found in Xiao et al.^9^, Lantican et al.^4^ and Yang et al.^28^.

Initially, 40,614 SNPs were identified. However, 53.97% of markers were not anchored to chromosomes and were not used in most subsequent analyses. However, once new versions of the coconut genome are improved, this larger set of SNPs can be used in future studies and represents a well of information available to breeders. Polymorphisms were widely distributed across the 16 chromosomes and were highly correlated with the length and number of genes on each chromosome. The transition/transversion ratio was consistent with that observed in other studies of palm species^42^. Transitions are usually more frequent than transversions and less likely to result in amino acid substitutions in protein-coding sequences and therefore they are more likely to persist in populations during natural selection^43^.

Because LD may affect the inference of the population structure, an LD < 0.2 filter was applied, which resulted in a decrease of the number of SNPs to 1,271. All approaches used in this study revealed there is a genetic structure in coconut populations in Colombia even though both coasts are geographically close to each other in northern south America. Structure analyses, principal component analysis and phylogenetic networks are all consistent with four groups explaining the genetic structure of these populations (Figs. 2, 3, 6). Genetic variation among coconut accessions based on molecular markers have been documented in previous studies; for a detailed review see Meerow et al.^14^. Zizumbo-Villareal and Arellano-Morin^44^ found genetic differences between Atlantic Tall and Pacific Tall coconuts, and a differentiation within Pacific tall coconuts. A study from the Philippines was able to cluster populations with similar genetic distances for morphological traits. A distinction between morphological and molecular markers was found for the Atlantic and Pacific gene pools^45^. Our results suggest that similarities at the phenotypic level do not imply close genetic relationship.

In Colombia, farmers lack knowledge of specific cultivars they are planting, and there is not a formal genetic breeding program developed for coconut in the country so far. Coconut accessions do not have a known origin and have not been identified at the genotypic level. Twenty-nine Atlantic coast accessions had a membership coefficient > 0.6 and very few of these SNPs were found in Pacific coast accessions (Figs. 2a, 3). They display characteristics of the Atlantic wild type described by Harries^3^ (Fig. 2a, Fig. S1). Average height ranges were higher (2.5–18.5 m) than palms growing in the Pacific coast (0.42 -14 m). Pacific coast accessions have a substructure represented in two main groups. The first cluster consisted of sixteen accessions with exclusive Pacific coast SNPs, and 29 accessions with a membership coefficient > 0.6. Their height ranges between 1.5 and 14 m (Fig. 3 and 6). A second cluster of four accessions with SNPs exclusive to the Pacific coast, and 16 accessions with a membership coefficient > 0.6, (Figs. 3, 6) had height ranges between 0.5 and 5 m (Figs. 3, 4, 5, 6). According to anecdotic comments made by farmers, this could be a dwarf genotype probably coming from southeast Asia.

Last, a third genotype was identified in our genetic structure analysis and consisted of only two admixed accessions found in both coasts (Figs. 3 and 6). A better characterization and identity of this genotype remains to be confirmed with a bigger sample size. Accessions with membership coefficients < 0.6 were considered hybrids, probably including commercial planting material, which is usually made up of dwarf × tall hybrids that have an intermediate semi-tall morphological phenotype^4,46^. Eleven recent hybrids were identified here, and they all were found in the Atlantic coast. We did not find any recent hybrids in the Pacific coast (Fig. S4). Introgressed individuals on the Atlantic coast involve the genotype recognized here as Pacific 2 most of the time. This suggests indirectly that they could be distinct from the Pre-Columbian Pacific Tall which is rare on the Atlantic coast.

Previously published studies^13,14,44,47^ found high heterozygosity rate in the Atlantic group. On the contrary, Pacific populations usually have been found to have a lower heterozygosity rate, because of a founder effect from coconut coming from Southeast Asia^1^. Putative Dwarfs should be almost entirely homozygous. Dwarfs have been suggested to originate from a tall phenotype that was domesticated in Southeast Asia and brought to other parts of the world recently^47^. They should mostly be those accessions with an IDB value closer to one (Fig. S5). In this study we found both gene pools have low nucleotide diversity, possibly indicating a strong bottleneck during cultivation of coconut in Colombia, which has drastically reduced diversity. This has also been shown by Gunn et al.^1^ and Kriswiyanti et al.^48^. Mean value of genetic diversity of Pacific coast coconuts found here, strengthens results found in previous studies that used a limited number of molecular markers such microsatellites^13,15,49–51^. Our study was based on many more molecular markers across the genome and might not be comparable to any other study of coconut presented so far because it was not possible to sample common material to other published work.

Estimation of the genome-wide distribution possesses the advantage of using many markers spread across the genome, as opposed to a candidate-gene screen for selection, particularly when the underlying demographic processes may not be well known in advance^52^. In our study, Tajima's D > 0 may suggest soft genetic sweeps that bring hitchhiker variants to high frequency, causing a population-wide reduction in the genetic diversity around the selected locus^53^. However, those regions may also occur within the genome because of random drift, and they are not distinguishable from regions that have undergone a selective sweep^21^. This could explain the low genetic diversity found in both Atlantic and Pacific Colombian coast gene pools. Our Fst results also suggest positive selection in the Pacific coast of Colombia accessions is favoring some alleles (Fig. 2c) whereas in the Atlantic coast accessions balancing selection maintains polymorphism over time^52^.

Linkage disequilibrium (LD) is the non-random association of alleles at different loci and is influenced by various factors. For instance, domestication, population subdivision, and selection can enhance LD in the genome^54^. Colombian Atlantic coast accessions reach half decay faster at 4 Mb than the Pacific coast ones at 17 Mb. The difference in half LD decay between the Atlantic and Pacific coast groups might be related to the different reproductive systems within *C*. *nucifera*, and to domestication and selective pressure preserving specific haplotype blocks^54^. LD can be affected by extreme genetic drift in domestication and breeding during evolution^55^. A relatively slow LD decay as the one we observed in coconut originating on the Pacific coast has been observed in other perennial or clonally propagated crops, such as artichoke^54^. The High proportion of SNPs with low LD decay for the Atlantic coast gene pool suggest genome-wide association study can be used to inform the breeding of the coconut varieties in Colombia. For this study LD was only measured based on half of the genome, and sequences used might not include regions on the genome with low recombination rates or with low polymorphisms. This might cause an overestimation of the average LD decay.

Phylogenetic networks represent conflicting signals in non-treelike processes, such as hybridization followed by introgression. The network displays relative evolutionary distances between taxa as well as uncertainty in the groupings in the form of “splits” of internal branches. Our results suggest high levels of hybridization, gene flow or incomplete lineage sorting among cultivars in Colombia (Fig. 6).

Further work could incorporate more wild individuals from the Pacific coast of Colombia and examine whether cultivated samples from the Pacific Islands present a continuum or a hard genetic discontinuity as might be expected with multiple domestication events.

Genotyping by sequencing data has proved useful and reliable for the identification of high-quality SNPs in coconut. We investigated genetic diversity, and structure of Colombian coconut populations. The pattern of variation found by genomic wide analysis indicates the existence of four groups of coconut populations in northern South America: the Pacific groups with a slow LD decay with low Fst and π, and the Atlantic one with slightly higher genetic diversity, fast LD decay and phenotypic similarity to the wild type of coconut populations. Information generated in this study will contribute to the knowledge of coconut genotypes and genetic diversity present in Colombia. Red ring disease in coconut and other cultivated and native palms in the Neotropics have reached epidemic levels. Knowledge of the genetic structure of Colombian coconut, including regions with augmented levels of genetic diversity, may ultimately prove useful in targeting source populations for disease resistance and other crop improvement traits. This work is a first step towards future genome-wide association mapping studies and the identification of SNP markers able to enhance the precision breeding for horticultural traits in cultivated coconut palms.

## Supplementary Information

Supplementary Information.

## Data Availability

All data have been deposited in Bioproject (PRJNA579494), SRA (SRR10345275-SRR10345401).

## Code availability

All code is available at https://github.com/TheAriasLab/POPULATIONS_GENOMICS.
