# Supplementary material for: Genome-wide diversity analysis to infer population structure and linkage disequilibrium among Colombian coconut germplasm
Source: Sci Rep. 2022 Feb 22;12:2958. doi: 10.1038/s41598-022-07013-w (PMC8863804; doi:10.1038/s41598-022-07013-w)
Supplement: Supplementary file 1 — Supplementary Information. [file 41598_2022_7013_MOESM1_ESM.pdf]

Genome-wide diversity analysis to infer population structure and linkage disequilibrium among Colombian coconut germplasm

Jorge Mario Muñoz-Pérez, Gloria Patricia Cañas, Lorena López and Tatiana Arias

Supplementary

Figure S1. Histogram of frequencies showing the depth of 40,614 SNPs aligned to the coconut genome and filtered to meet quality parameters required by the software Structure (Hubisz et al. 2009).

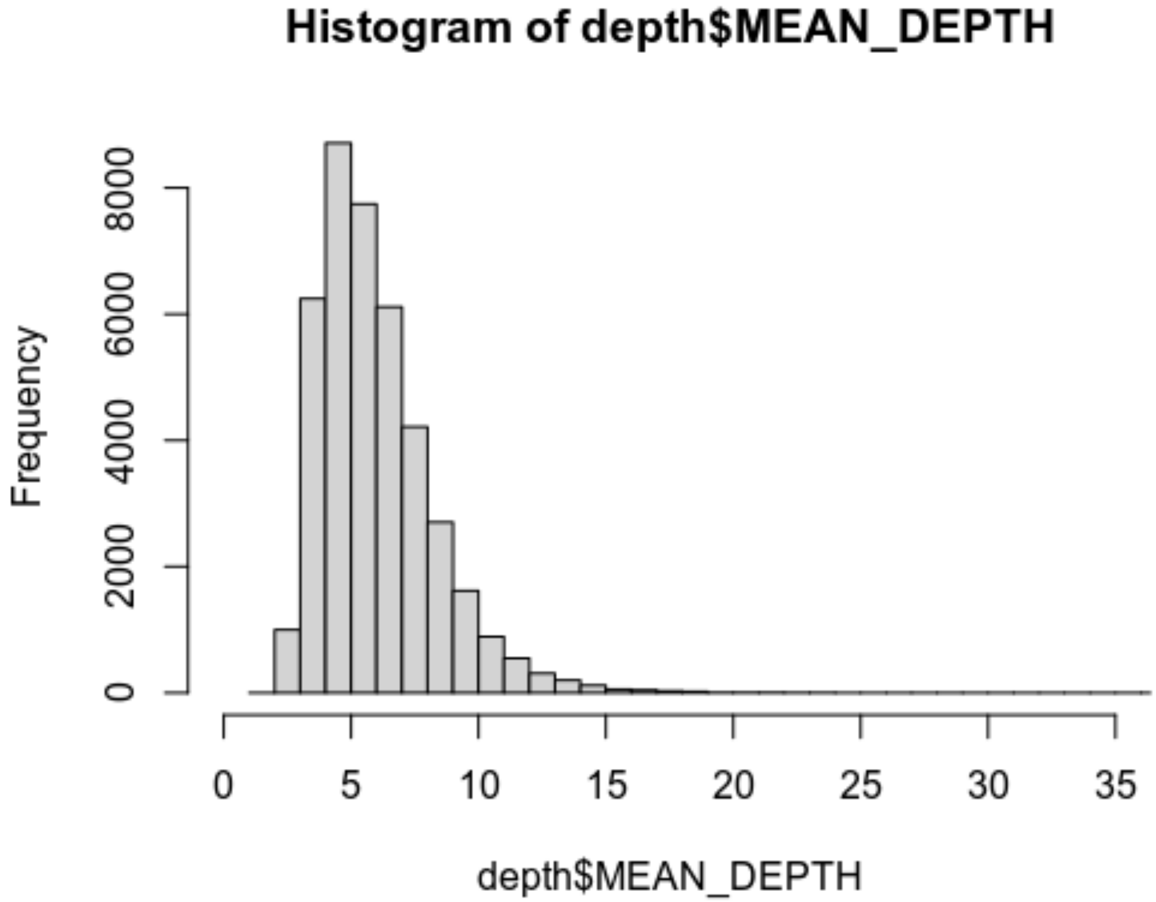

Figure S2. Results showed when the method of Evanno et al. 2005 was used to calculate delta K as a measure that best describes the number of clusters in the data. Here an analysis of K=2 to K=6 showed the highest delta K was K=4.

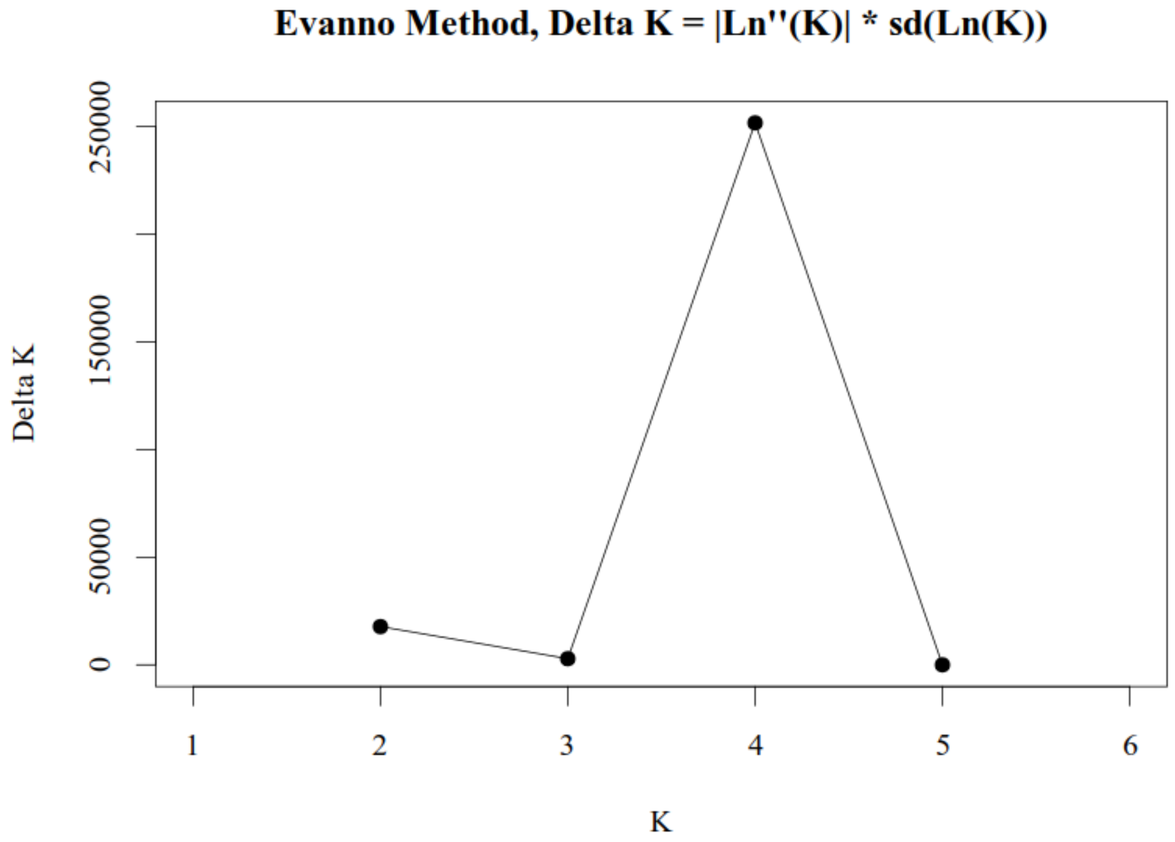

Figure S3. Analysis of the population structure using 112 accessions belonging to the Colombian coconut diversity panel with K = 2, K = 3. K= 5, K= 6.

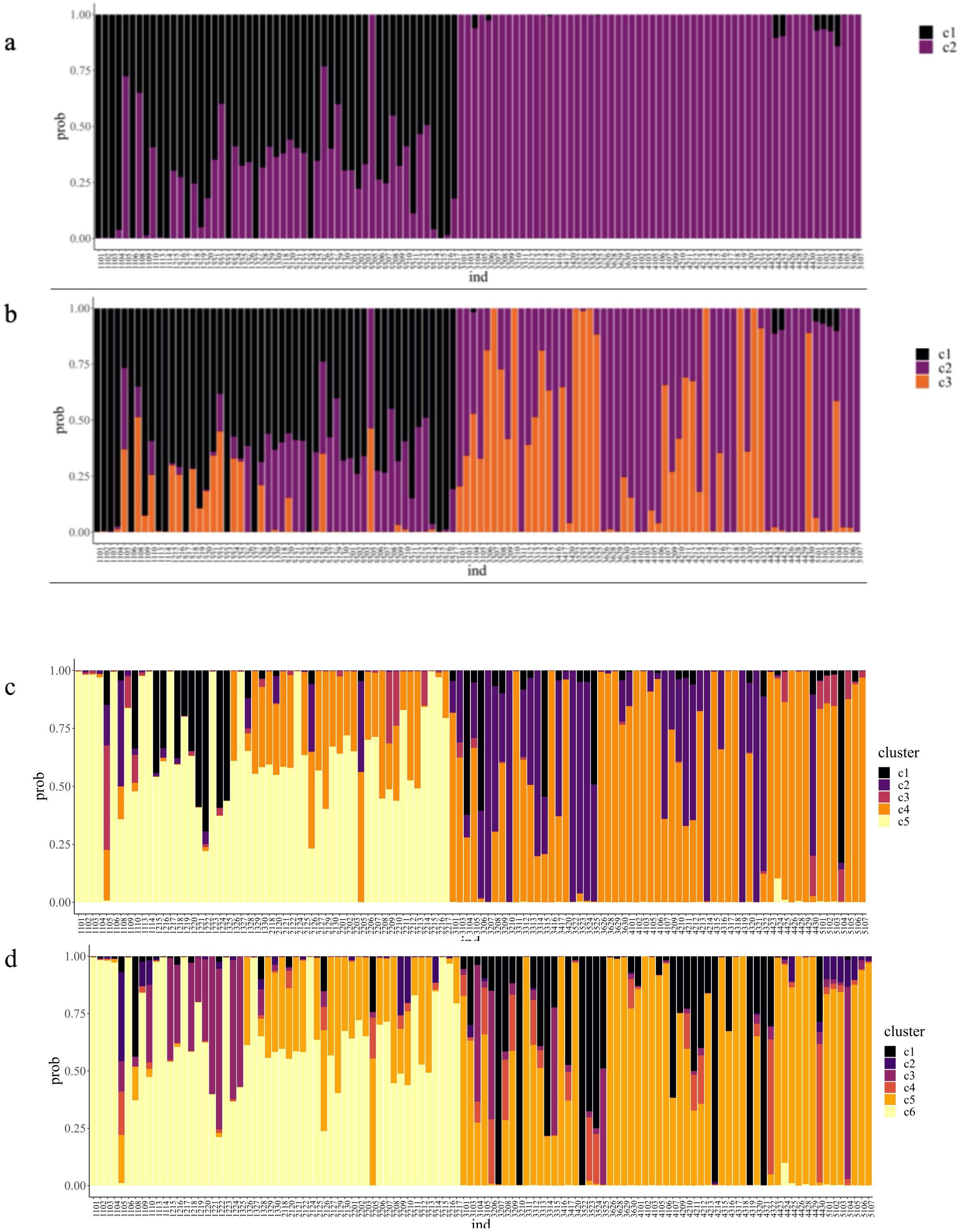

Figure S4. Recent hybrids were detected using Snapclust from the Adegenet R package V2.1.3. (Jombart 2008).

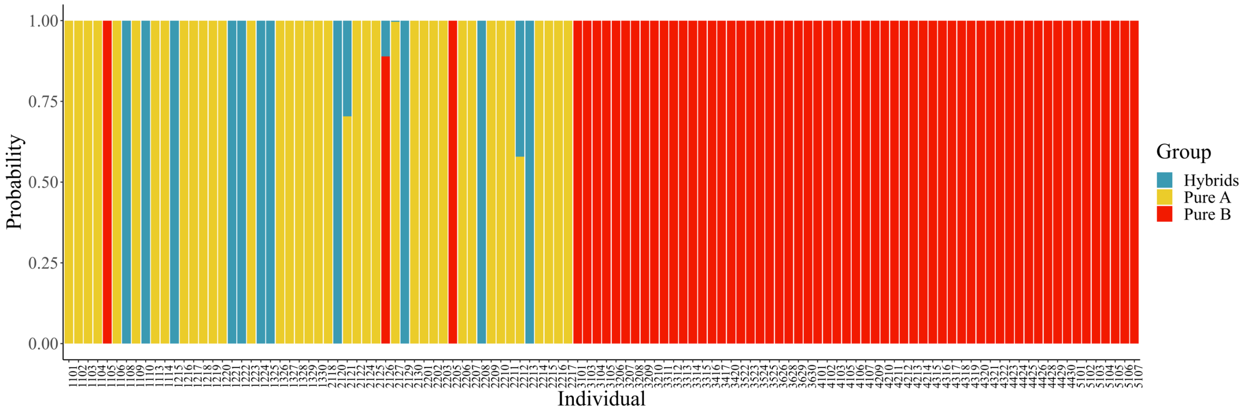

Figure S5. The kinship coefficient for Identity By Descent (IBD) was calculated done using the PLINK v1.90b5.2, Method of Moment (MoM) (Purcell et al. 2007).

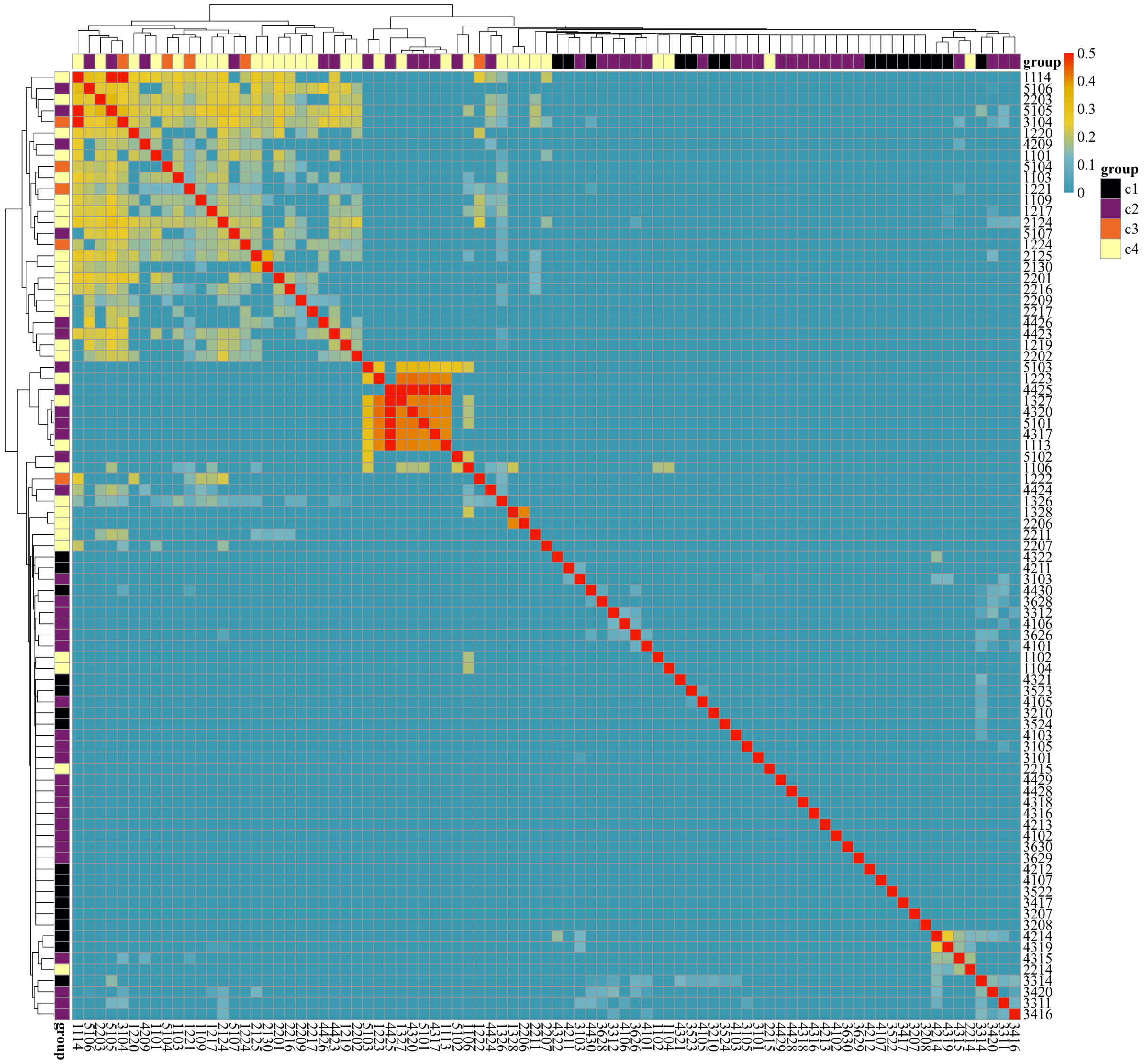

Table S1. Voucher, geographic coordinates for northern South America coconut accessions, when available.

ind	Department	Municipality	Locality	Latitud	Logitud	Coast
**1101**	Antioquia	San Juan de Urabá	Uveros	8.4975	76.705	Atlantic
**1102**	Antioquia	San Juan de Urabá	Uveros	8.46694	76.60417	Atlantic
**1103**	Antioquia	San Juan de Urabá	Uveros	8.49639	76.80389	Atlantic
**1104**	Antioquia	San Juan de Urabá	Uveros	8.475	76.79778	Atlantic
**1105**	Antioquia	San Juan de Urabá	Uveros	8.45583	76.59028	Atlantic
**1106**	Antioquia	San Juan de Urabá	Uveros	8.42111	76.62889	Atlantic
**1107**	Antioquia	San Juan de Urabá	Uveros	8.37306	76.62944	Atlantic
**1108**	Antioquia	San Juan de Urabá	Uveros	8.46444	76.74806	Atlantic
**1109**	Antioquia	San Juan de Urabá	Uveros	8.26861	76.74861	Atlantic
**1110**	Antioquia	San Juan de Urabá	Uveros	8.35333	76.69806	Atlantic
**1111**	Antioquia	San Juan de Urabá	Uveros	8.43056	76.84056	Atlantic
**1112**	Antioquia	San Juan de Urabá	Uveros	8.28917	76.57222	Atlantic
**1113**	Antioquia	San Juan de Urabá	Uveros	8.51361	76.82083	Atlantic
**1114**	Antioquia	San Juan de Urabá	Uveros	8.45083	76.82389	Atlantic
**1215**	Antioquia	San Juan de Urabá	La Balsilla	8.43222	76.76528	Atlantic
**1216**	Antioquia	San Juan de Urabá	La Balsilla	8.40806	76.82722	Atlantic
**1217**	Antioquia	San Juan de Urabá	La Balsilla	8.35389	76.82667	Atlantic
**1218**	Antioquia	San Juan de Urabá	La Balsilla	8.31639	76.82361	Atlantic
**1219**	Antioquia	San Juan de Urabá	La Balsilla	8.29583	76.76167	Atlantic
**1220**	Antioquia	San Juan de Urabá	La Balsilla	8.31778	76.72833	Atlantic
**1221**	Antioquia	San Juan de Urabá	La Balsilla	8.34222	76.735	Atlantic
**1222**	Antioquia	San Juan de Urabá	La Balsilla	8.26972	76.72861	Atlantic
**1223**	Antioquia	San Juan de Urabá	La Balsilla	8.4725	76.75722	Atlantic
**1224**	Antioquia	San Juan de Urabá	La Balsilla	8.43472	76.67972	Atlantic
**1326**	Antioquia	Arboletes	El Destino	8.41167	76.52861	Atlantic
**1327**	Antioquia	Arboletes	El Destino	8.44778	76.47972	Atlantic
**1328**	Antioquia	Arboletes	El Destino	8.47306	76.51361	Atlantic
**1329**	Antioquia	Arboletes	El Destino	8.53583	76.48694	Atlantic
**2117**	Córdoba	Moñitos	Behiacoita	8.47472	76.73389	Atlantic
**2118**	Córdoba	Moñitos	Pueblito	8.62194	75.93167	Atlantic
**2119**	Córdoba	Moñitos	Pueblito	8.5375	75.89944	Atlantic
**2120**	Córdoba	Moñitos	Pueblito	8.53028	76.03111	Atlantic
**2121**	Córdoba	Moñitos	Pueblito	8.44083	75.96722	Atlantic
**2122**	Córdoba	Moñitos	Pueblito	8.67833	75.95472	Atlantic
**2123**	Córdoba	Moñitos	Pueblito	8.59806	75.80333	Atlantic
**2124**	Córdoba	Moñitos	Pueblito	8.67556	75.76806	Atlantic

**2125**	Córdoba	Moñitos	La Rada	8.53028	76.89194	Atlantic
**2126**	Córdoba	Moñitos	La Rada	8.73333	76.69278	Atlantic
**2128**	Córdoba	Moñitos	Behiacoita	8.7	76.73417	Atlantic
**2129**	Córdoba	Moñitos	El Destino	8.60639	76.83417	Atlantic
**2130**	Córdoba	Moñitos	El Destino	8.55611	76.74389	Atlantic
**2201**	Córdoba	Puerto Escondido	Mucuna	8.17417	76.04556	Atlantic
**2202**	Córdoba	Puerto Escondido	Mucuna	8.17417	76.04556	Atlantic
**2203**	Córdoba	Puerto Escondido	Marimar	8.33472	76.27806	Atlantic
**2204**	Córdoba	Puerto Escondido	Marimar	8.86861	76.29417	Atlantic
**2205**	Córdoba	Puerto Escondido	Marimar	8.28944	76.26694	Atlantic
**2206**	Córdoba	Puerto Escondido	El Paraíso	8.34639	76.11667	Atlantic
**2207**	Córdoba	Puerto Escondido	El Paraíso	8.26694	76.23083	Atlantic
**2209**	Córdoba	Puerto Escondido	La Unión	8.34528	76.13	Atlantic
**2210**	Córdoba	Puerto Escondido	La Unión	8.13861	76.20028	Atlantic
**2211**	Córdoba	Puerto Escondido	Monserrate	8.76944	76.19389	Atlantic
**2212**	Córdoba	Puerto Escondido	Monserrate	8.68222	76.16972	Atlantic
**2213**	Córdoba	Puerto Escondido	La Maravilla	8.8225	76.16972	Atlantic
**2214**	Córdoba	Puerto Escondido	La Maravilla	8.81778	76.14194	Atlantic
**2215**	Córdoba	Puerto Escondido	La Maravilla	8.66639	76.1375	Atlantic
**2216**	Córdoba	Puerto Escondido	Porto alegre	8.68583	76.22417	Atlantic
**2217**	Córdoba	Puerto Escondido	Porto alegre	8.695	76.215	Atlantic
**3101**	Nariño	Tumaco	San José del Guayabo	2.2375	77.99083	Pacific
**3102**	Nariño	Tumaco	San José del Guayabo	2.2525	77.98944	Pacific
**3103**	Nariño	Tumaco	San José del Guayabo	2.21889	77.99444	Pacific
**3104**	Nariño	Tumaco	San José del Guayabo	2.20417	77.99972	Pacific
**3105**	Nariño	Tumaco	San José del Guayabo	2.18861	78.04972	Pacific
**3206**	Nariño	Tumaco	Tablón dulce	2.39333	77.99028	Pacific
**3207**	Nariño	Tumaco	Tablón dulce	2.41222	78.00083	Pacific
**3209**	Nariño	Tumaco	Tablón dulce	2.33194	78.04861	Pacific

**3210**	Nariño	Tumaco	Tablón dulce	2.30583	77.925	Pacific
**3311**	Nariño	Tumaco	Chagui	2.34528	78.08111	Pacific
**3312**	Nariño	Tumaco	Chagui	2.46972	78.12944	Pacific
**3313**	Nariño	Tumaco	Chagui	2.52694	77.935	Pacific
**3314**	Nariño	Tumaco	Chagui	2.38667	78.05361	Pacific
**3315**	Nariño	Tumaco	Chagui	2.32389	78.06528	Pacific
**3416**	Nariño	Tumaco	Buenos Aires	2.37139	78.13278	Pacific
**3417**	Nariño	Tumaco	Buenos Aires	2.4775	77.91361	Pacific
**3418**	Nariño	Tumaco	Buenos Aires	2.50528	78.11333	Pacific
**3419**	Nariño	Tumaco	Buenos Aires	2.37583	77.9525	Pacific
**3420**	Nariño	Tumaco	Buenos Aires	2.36472	77.9525	Pacific
**3521**	Nariño	Tumaco	Rosario	2.23861	78.05056	Pacific
**3522**	Nariño	Tumaco	Rosario	2.2825	78.15056	Pacific
**3523**	Nariño	Tumaco	Rosario	2.30611	78.21472	Pacific
**3524**	Nariño	Tumaco	Rosario	2.245	78.295	Pacific
**3525**	Nariño	Tumaco	Rosario	2.25028	78.305	Pacific
**3617**	Nariño	Tumaco	Gualayo	2.3025	78.23972	Pacific
**3626**	Nariño	Tumaco	Gualayo	2.30417	78.25194	Pacific
**3628**	Nariño	Tumaco	Gualayo	2.30917	77.99583	Pacific
**3629**	Nariño	Tumaco	Gualayo	2.37139	78.24472	Pacific
**3630**	Nariño	Tumaco	Gualayo	2.35722	78.25361	Pacific
**4101**	Cauca	Guapi	Playa Blanca	3.11583	77.19389	Pacific
**4102**	Cauca	Guapi	Playa Blanca	3.10583	77.20361	Pacific
**4103**	Cauca	Guapi	Playa Blanca	3.17778	77.22639	Pacific
**4104**	Cauca	Guapi	Playa Blanca	3.18417	77.26	Pacific
**4105**	Cauca	Guapi	Playa Blanca	3.25917	77.25556	Pacific
**4106**	Cauca	Guapi	Playa Blanca	3.13944	77.25222	Pacific
**4107**	Cauca	Guapi	Playa Blanca	3.23556	77.27056	Pacific
**4209**	Cauca	Guapi	Quiroga	3.16028	77.30056	Pacific
**4210**	Cauca	Guapi	Quiroga	3.11722	77.26222	Pacific
**4211**	Cauca	Guapi	Quiroga	3.33917	77.34806	Pacific
**4212**	Cauca	Guapi	Quiroga	3.27778	77.17306	Pacific
**4213**	Cauca	Guapi	Quiroga	3.29222	77.17639	Pacific

**4214**	Cauca	Guapi	Quiroga	3.09028	77.23972	Pacific
**4316**	Cauca	Guapi	Preba	3.2825	77.49667	Pacific
**4317**	Cauca	Guapi	Preba	3.27167	77.49167	Pacific
**4318**	Cauca	Guapi	Preba	3.265	77.29361	Pacific
**4319**	Cauca	Guapi	Preba	3.06694	77.40639	Pacific
**4320**	Cauca	Guapi	Preba	3.05389	77.38	Pacific
**4321**	Cauca	Guapi	Preba	3.06722	77.44222	Pacific
**4322**	Cauca	Guapi	Preba	3.28611	77.48639	Pacific
**4417**	Cauca	Guapi	Obregón	3.28167	77.23111	Pacific
**4423**	Cauca	Guapi	Obregón	3.2575	77.40194	Pacific
**4424**	Cauca	Guapi	Obregón	3.32528	77.42556	Pacific
**4425**	Cauca	Guapi	Obregón	3.35056	77.395	Pacific
**4428**	Cauca	Guapi	Obregón	3.28972	77.45528	Pacific
**4429**	Cauca	Guapi	Obregón	3.30139	77.35611	Pacific
**4430**	Cauca	Guapi	Obregón	3.19167	77.25417	Pacific
**5101**	Choco	Nuqui	Coqui	5.67139	77.46639	Pacific
**5102**	Choco	Nuqui	Coqui	5.68	77.47056	Pacific
**5103**	Choco	Nuqui	Coqui	5.68222	77.47333	Pacific
**5104**	Choco	Nuqui	Coqui	5.67861	77.4625	Pacific
**5105**	Choco	Nuqui	Coqui	5.68667	77.44639	Pacific
**5106**	Choco	Nuqui	Coqui	5.7	77.41	Pacific
**5107**	Choco	Nuqui	Coqui	5.70361	77.40361	Pacific

Table S2. Enzymes used in a pilot GBS analysis.

<table>
<tr><td colspan="4">SNPs per enzyme test</td></tr>
<tr><td>Enzyme</td><td># SNPs</td><td></td><td></td></tr>
<tr><td>Pstl-MstI</td><td>1420</td><td></td><td>SNPs discovery using Freebayes v1.0.2-16</td></tr>
<tr><td>MslI</td><td>44655</td><td></td><td>SNP filtering according to GBS rules: (depth 8 reads min, SNPs found min in 8 individuals out of 12 , MAF > 0.1</td></tr>
<tr><td colspan="4">Reads per enzyme test</td></tr>
<tr><td>Enzyme</td><td>Restriction site</td><td>No. reads</td><td></td></tr>
<tr><td>MslI</td><td>5'...CAYNN|NNRTG...3'</td><td>127,899,980</td><td></td></tr>
<tr><td>3'...GTRNN|NNYAC...5'</td><td>127899980</td><td></td><td></td></tr>
<tr><td>PstI-MspI</td><td>5'...C|CGG...3'</td><td>122,593,202</td><td></td></tr>
<tr><td>3'...GGC|C...5'</td><td>122593202</td><td></td><td></td></tr>
</table>
